# Contribution of sleep to the repair of neuronal DNA double-strand breaks: evidence from flies and mice

**DOI:** 10.1038/srep36804

**Published:** 2016-11-10

**Authors:** Michele Bellesi, Daniel Bushey, Mattia Chini, Giulio Tononi, Chiara Cirelli

**Affiliations:** 1 Department of Psychiatry, University of Wisconsin-Madison, 6001 Research Park Blvd, Madison, WI 53719, USA; 2 Department of Experimental and Clinical Medicine, Section of Neuroscience and Cell Biology, Università Politecnica delle Marche, Ancona, Italy

## Abstract

Exploration of a novel environment leads to neuronal DNA double-strand breaks (DSBs). These DSBs are generated by type 2 topoisomerase to relieve topological constrains that limit transcription of plasticity-related immediate early genes. If not promptly repaired, however, DSBs may lead to cell death. Since the induction of plasticity-related genes is higher in wake than in sleep, we asked whether it is specifically wake associated with synaptic plasticity that leads to DSBs, and whether sleep provides any selective advantage over wake in their repair. In flies and mice, we find that enriched wake, more than simply time spent awake, induces DSBs, and their repair in mice is delayed or prevented by subsequent wake. In both species the repair of irradiation-induced neuronal DSBs is also quicker during sleep, and mouse genes mediating the response to DNA damage are upregulated in sleep. Thus, sleep facilitates the repair of neuronal DSBs.

Sleep is a pervasive and universal behavior. It occupies a third of human life and is present in every animal species that has been carefully studied^1^. While it is common experience that the brain needs sleep to function properly, and that wake is a costly state that cannot be sustained indefinitely, the reasons why sleep is needed remain elusive. The synaptic homeostasis hypothesis (SHY) states that sleep is required to reestablish “synaptic homeostasis”, which is challenged by the remarkable plasticity of the brain^2^,^3^,^4^. SHY predicts that during wake synaptic connections throughout the brain undergo potentiation, while we learn new facts and regularities about the environment, resulting in a net increase in synaptic strength in many brain regions. The remarkable and pervasive plasticity of the brain is essential for survival but is also costly at the cellular and at the systems level, because stronger synapses increase the demand for energy and cellular supplies, lead to decreases in the signal-to-noise ratio, and saturate the ability to learn. During sleep, while the brain is disconnected from the environment, neural circuits can be reactivated in an off-line mode and, according to SHY, a systematic renormalization of synaptic strength can occur.

Molecular, electrophysiological and structural markers of synaptic strength are higher after wake than after sleep, including the synaptic expression of excitatory GluA1-containing AMPA receptors, the amplitude and slope of cortical evoked responses, the amplitude and frequency of miniature excitatory synaptic potentials, and the size and/or number of synaptic terminals (reviewed in ref. 4). At the cellular level, there are several reasons why the wake-related increase in synaptic strength is costly. One reason is the high energy consumption required to sustain synaptic activity^5^, which is expected to increase with increasing synaptic strength in wake, resulting in higher mitochondrial activity during sustained wake relative to sleep^6^,^7^,^8^,^9^. In the cerebral cortex, oxidants production linked to mitochondrial activity increases during extended wake with exploration^10^, and wake-related metabolic activation puts locus coeruleus noradrenergic cells, and other wake-promoting neurons, at high risk of oxidative damage^11^,^12^. Another cost associated with synaptic strengthening stems from the need for new cellular components, including receptors and other structural proteins, which then need to be maintained *in situ*^13^,^14^. Extended wake consistently triggers the unfolded protein response, which slows down the synthesis of most proteins^15^,^16^,^17^, thus creating an imbalance between increased need for supplies and impaired ability to produce them. Finally, extracellular glutamate levels increase in the course of wake^18^, due to higher overall neuronal activity and/or lower clearance of solutes from the interstitial space in wake relative to sleep^19^, possibly increasing the risk for glutamate-induced excitotoxicity.

It was recently found that physiological brain activity associated with exploration of a novel environment induces DNA double-strand breaks (DSBs) in neurons of young adult wild-type mice^20^, a puzzling result since DSBs may lead to cell death unless they are promptly repaired. The number of cells displaying DSBs foci returned to baseline levels after mice were allowed to recover in their home cages for 24 hours^20^, but their sleep/wake behavior was not monitored. Other studies found that the transient appearance of DSBs appears to be an obligatory, type 2 topoisomerase-dependent step during the induction of several immediate-early genes, many of which have been implicated in synaptic plasticity^21^,^22^. These intriguing results prompted us to ask whether it is wake, or specifically wake conditions conducive to learning and synaptic plasticity, which lead to the formation of DSBs, and whether sleep provides any selective advantage over wake during their repair. We addressed these questions in both flies and mice, because wake plasticity and sleep need are strongly linked in both species^23^,^24^. Moreover, we investigated the role of sleep after genotoxic stress caused by a non-lethal dose of whole body gamma irradiation, which induces a massive number of DSBs through a mechanism independent of type 2 topoisomerase. We find that in both the fly and the mouse brain time spent in exploratory behavior, more than simply time spent awake, induces DSBs and in mice, the repair of exploration-induced DSBs is delayed or prevented by subsequent wake. Moreover, in both species the repair of irradiation-induced neuronal DSBs is faster during sleep.

## Results

### Wake with exploration induces DSBs in the fly brain

**Figure 1:**
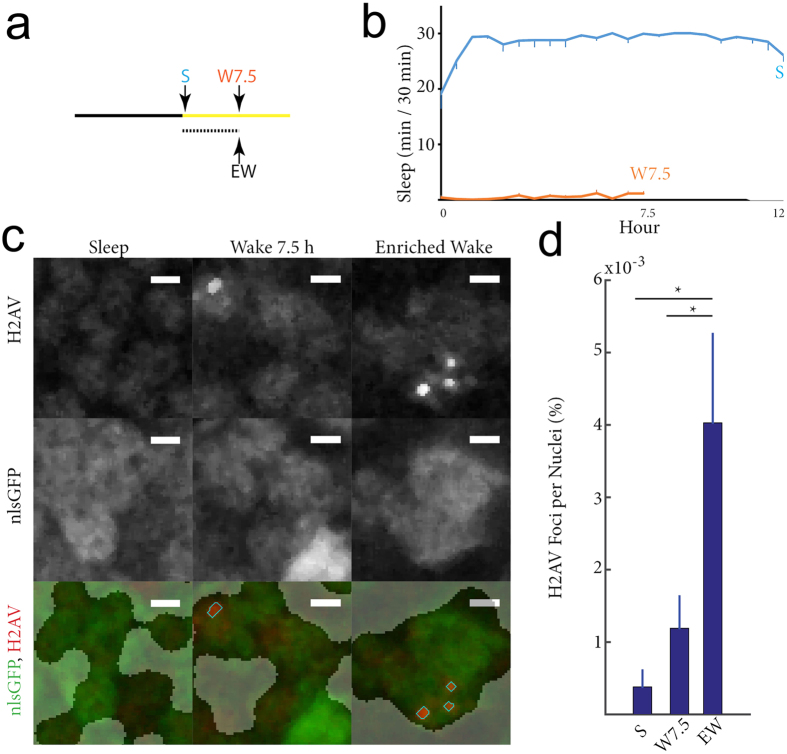
DSBs accumulate in the fly brain after enriched wake. (**a**) The 3 experimental groups collected after the 12 h dark period (black bar) and during the light period (yellow bar). (**b**) Sleep amount before collection is shown for consecutive 30 min periods in the 2 groups housed in single tubes. (**c**) Representative images from a single layer within the mushroom body cells. Intensity from H2AV (top) and nlsGFP (middle) and combined (bottom) H2AV (red) and nlsGFP (green) signals are shown. Scale bar = 1 μm. In the bottom image, opaque areas indicate the cells whose background H2AV and nlsGFP signals are below the threshold set to identify neuronal nuclei (see Methods for detail). Outlined in light blue are the regions that were counted as H2AV foci because their signal intensity was >30 times background levels and their volume was >0.2 um^3^. (**d**) Percentage of nuclei across the fly brain containing H2AV foci in S (n = 14), W7.5 (n = 20) and EW (n = 8) flies.*p < 0.05 with Kruskal-Wallis test followed by Mann-Whitney test. Bars indicate standard error.

Three groups of flies were collected (Fig. 1a), all including only females because their sleep/wake cycle is more consolidated, i.e. females remain awake most of the day and sleep most of the night, while males also tend to “nap” in the early afternoon^25^. Flies in 2 of the groups were housed in single tubes (no enrichment), and thus the behavior of each individual animal could be monitored continuously: S flies spent the 12 h of the night mostly asleep, while W7.5 flies spent the first ~7.5 h of the day mostly awake (Fig. 1b). Previous evidence shows that 7.5 h represent an interval of time long enough to detect differences in DSBs formation and repair^26^. The third group (EW) was collected at the same circadian time as the W7.5 group, but these flies spent the first ~7.5 h of the day in an enriched environment that consisted of large tubes containing 25–30 individuals; in these large tubes social interactions, climbing, and short flights were possible. Although the behavior of each EW fly could not be monitored, we assume that they were mostly awake during the day, as W7.5 flies were, and previous experiments found that this was indeed the case^27^. DSBs were identified in the fly brain using an antibody that specifically binds to phosphorylated gamma H2AV^28^. Phosphorylation on the histone H2A tail occurs rapidly at DSB sites in both Drosophila (variant H2AV) and in mammals (variant H2AX), providing a conserved and specific marker of this type of DNA damage. Neuronal nuclei were marked using a pan-neuronal GAL4 driver that drove expression of the GFP transgene fused to a nuclear localization signal (nlsGFP). Strongly staining H2AV puncta corresponding to DSBs tended to increase after wake relative to sleep, but the difference was not significant when comparing S flies to W7.5 flies (p = 0.53). By contrast, a significant increase in DSBs was found between EW and S flies (p = 0.0004) and between EW and W7.5 flies (p = 0.0019), suggesting that enriched wake, more than wake per se, is associated with DSBs formation (Fig. 1c,d).

### Sleep boosts neuronal DSBs repair after whole body gamma-irradiation in female flies

**Figure 2:**
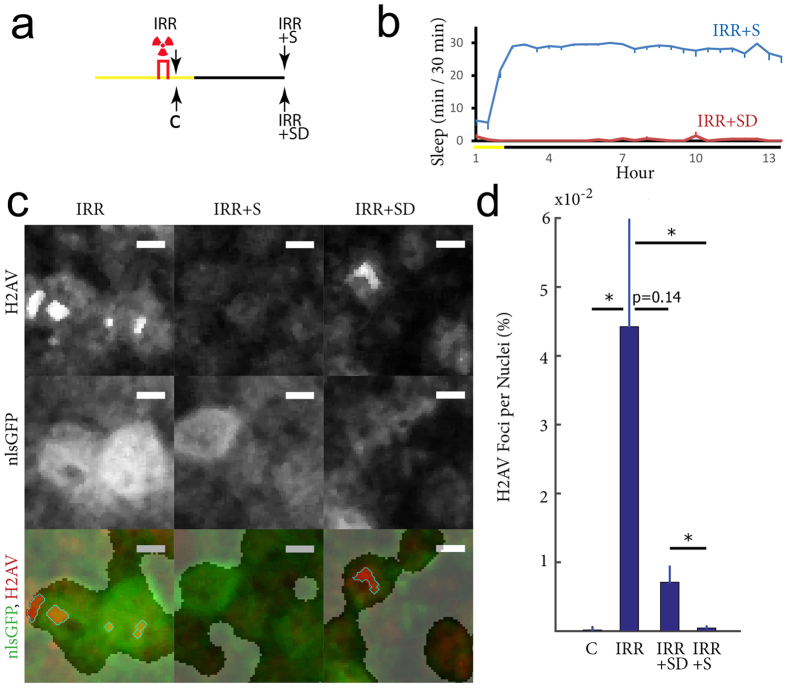
DSBs repair post-irradiation is facilitated by sleep in female flies. (**a,b**) The 4 experimental groups and the amount of sleep before collection in IRR + S and IRR + SD flies. The amount of sleep (min) is shown for consecutive 30 min periods. Yellow and black bars indicate day and night, respectively. (**c**) Representative images from single layers containing mushroom body cells. Intensity from the H2AV (top), nslGFP (middle) and combined (bottom, nslGFP green, H2AV red) are shown. Scale bar = 1 μm. Bottom row, clear areas are the nuclei, opaque areas are the non nuclear regions, i.e. regions whose background H2AV and nlsGFP signal is below the threshold set for nuclei (see Methods for detail). Outlined with a light blue contour are H2AV foci whose H2AV/background H2AV ratio exceeds threshold, and whose size is >0.2 um^3^. (**d**) Percentage of nuclei containing H2AV foci across the fly brain in a non-irradiated control group (C, n = 10), IRR (n = 9), IRR + S (n = 10), and IRR + SD (n = 6) flies. *p < 0.05 with Kruskal-Wallis test followed by Mann-Whitney test. Bars indicate standard error.

To test the effect of sleep on DSBs repair female flies were subjected to whole body gamma-irradiation and then were either allowed to sleep or sleep deprived throughout the dark period, when flies normally sleep (Fig. 2a,b). As expected many DSBs were caused by irradiation, although there was significant variability across flies. When irradiation was followed by sleep, DSB frequency declined significantly and in fact, returned to pre-irradiated levels (IRR + S *vs* IRR: p = 0.00002; IRR + S *vs* C: p = 0.64; Fig. 2c,d). After irradiation was followed by sleep deprivation, DSBs still greatly declined, but their number was not significantly different from the number seen immediately after irradiation (IRR + SD *vs* IRR, p = 0.14), while it was significantly higher than the number observed after sleep (IRR + SD *vs* IRR + S: p = 0.0007) or in the non-irradiated control (IRR + SD *vs* C: p = 0.0075).

### Wake with exploration induces DSBs in the mouse cerebral cortex and their repair is impaired by wake

**Figure 3:**
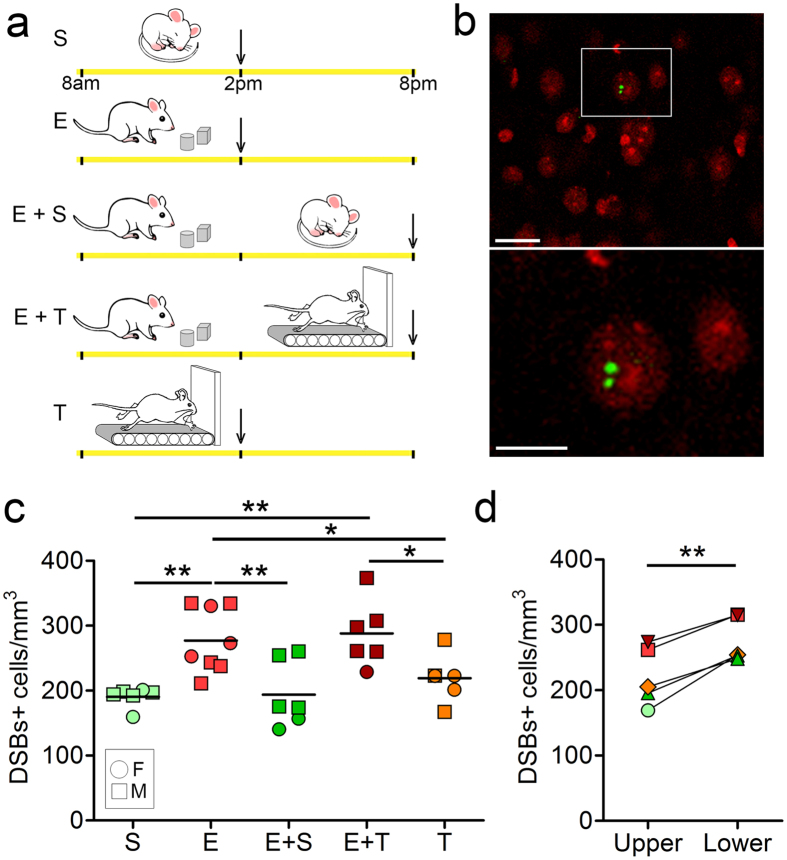
Effects of sleep and wake on DSBs in mouse frontal cortex. (**a**) Experimental design. The yellow bar indicates the 12 h light period (8 am–8 pm). Vertical arrows show the time of brain collection. S, sleep; E, exploration; T, treadmill. (**b**) Examples of nuclear localization of γH2AX positive foci (green) in immunostained sections of frontal cortex in an E mouse. Nuclei are labeled with propidium iodide (PI, red). Scale bars are 10 and 5 μm, respectively. (**c**) Number of cells containing 1 or more γH2AX positive foci per mm^3^ of frontal cortex in each of the 5 experimental groups. Each symbol is one mouse (M, male; F, female). (**d**) Average number of cells with γH2AX positive foci for each experimental group, shown separately for the upper and lower layers of frontal cortex. *p < 0.05; **p < 0.01.

We measured DSBs in the frontal cortex of mice belonging to 5 different experimental groups, all collected during the light period: sleep (S, 6 h), exploration (E, 6 h), exploration followed by sleep (E 6 h + S 6 h), exploration followed by wake on a treadmill with little/no exploration (E 6 h + T 6 h), and wake on a treadmill (T 6 h) (Fig. 3a). The early response to DSBs includes the phosphorylation of H2AX, a variant form of the histone H2A. DSBs were thus identified using an antibody that recognizes the phosphorylated histone protein H2A variant X at serine 139, a previously validated method to detect DSBs in mammalian brain cells^29^. As expected γH2AX positive foci were clearly visible within the nucleus, allowing positive cells to be identified and scored (Fig. 3b). We focused on the frontal cortex because the slow wave activity (SWA) of NREM sleep is largest in frontal areas in both humans and rodents. SWA increases with wake duration and declines during sleep, suggesting that it may reflect the need for sleep. SWA is also a marker of sleep intensity, because arousal thresholds during sleep are higher when SWA is higher^30^. Thus, frontal cortex may accumulate the largest need for sleep, may be uniquely sensitive to the effects of sleep deprivation, and/or the changes in neuronal activity associated with the occurrence of slow waves may be most pronounced in this area. Quantification of cells showing one or more DSB foci revealed differences among the 5 groups (one-way ANOVA, F = 7.3, p = 0.0004). Specifically, E showed a higher number of DSBs + cells than S in frontal cortex ( + 45.5 ± 25.9%, Newman-Keuls Multiple Comparison Test post-hoc test, q = 5.24, p < 0.01), confirming that exploration is associated with increased formation of DSBs in the cerebral cortex^20^. Moreover, we found that the occurrence of sleep after exploration reduced the number of DSBs + cells to the level seen in S animals (+1.6 ± 26.5%, Newman-Keuls Multiple Comparison Test post-hoc test, S *vs*. E + S, q = 0.18, p > 0.05) and, crucially, lower than in E mice (Newman-Keuls Multiple Comparison Test post-hoc test, q = 5.05, p < 0.01) and in E + T mice, whose brain was collected at the same time of day as the E + S mice but that spent the last 12 h awake (Newman-Keuls Multiple Comparison Test post-hoc test, E + S *vs*. E + T, q = 5.3, p < 0.01; Fig. 3c). In addition, E and E + T mice showed comparable numbers of DSBs + cells (q = 0.66, p > 0.05), despite the fact that E + T mice were awake for 6 additional hours, suggesting that as in flies, the induction of DSBs is mainly driven by the time spent in learning and exploratory behavior, rather than simply by the time spent awake. The role of exploration was further confirmed by the analysis of T mice, awake but without exposure to novel objects, whose number of DSBs + cells was significantly lower than in E mice (q = 3.496, p < 0.05) and in E + T mice (q = 3.889, p < 0.05), but not different from the number in S mice (q = 1.628, p > 0.05) and E + S mice (q = 0.1, p > 0.05). Pooling together DSBs values within each sex group, no significant differences were found between males and females (Fig. 3c, p = 0.34). By contrast, in all groups consistent differences were found across cortical layers, with deep layers (layers V-VI) showing greater density of DSBs than upper layers (layers II-III) (+25.3 ± 15.7%, paired t-test, t = 7.06, p = 0.002, Fig. 3d).

### Sleep boosts neuronal DSBs repair after whole body gamma-irradiation in male mice

**Figure 4:**
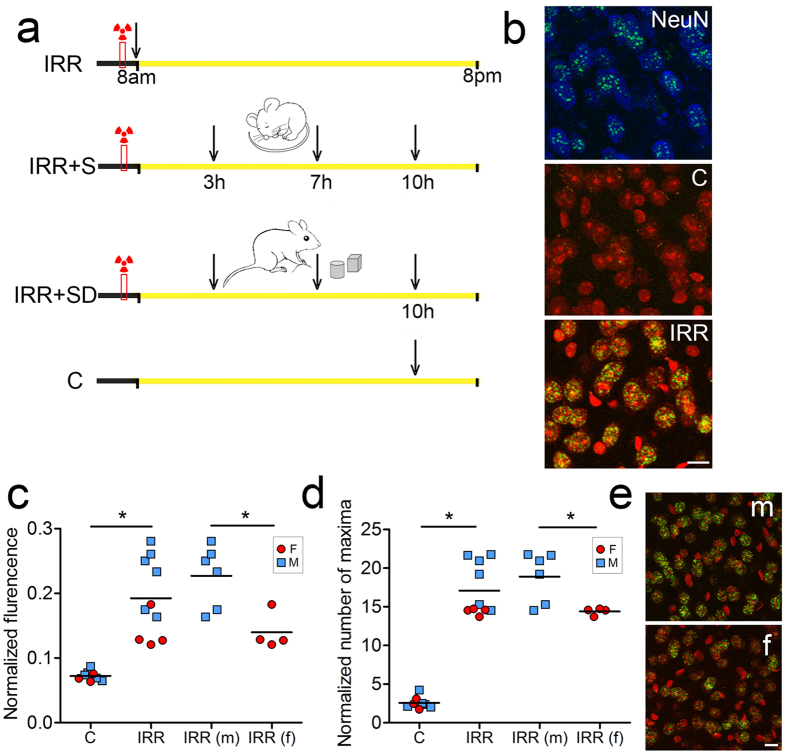
Effects of gamma-irradiation on DSBs in mouse frontal cortex. (**a**) Experimental design. The red box indicates the time when gamma-irradiation occurred, between 7 and 8 am, before light on. The yellow bar indicates the 12 h light period (8 am–8 pm). Vertical arrows show the time of brain collection. IRR, irradiation; S, sleep; SD, sleep deprivation; C, control. (**b**) Top panel: γH2AX positive foci (green) induced by gamma-irradiation were found primarily in neurons immunostained with the specific neuronal marker NeuN + (blue). Middle and bottom panels: representative images from the frontal cortex of a control mouse (C, middle panel) and a mouse killed within 1 hour post-irradiation (IRR, bottom panel). Scale bar = 10 μm. (**c,d**) Quantification of γH2AX normalized fluorescence and local maxima in C and IRR mice. Circle and square symbols indicate males and females, respectively. *p < 0.05 (**e**) Representative microscopic fields from the frontal cortex of a post γ-irradiated male (m) and a post γ-irradiated female (f) mouse showing the different number of γH2AX positive foci. Scale bar = 10 μm.

To further assess the role of sleep in DNA repair we studied 8 additional groups of mice (Fig. 4a), including a control group (C, no-irradiation) and 7 groups in which first we artificially induced a much higher number of DSBs through a very different mechanism, whole body gamma-irradiation. After this treatment, brains were collected within 1 hour (IRR), or after the mice were allowed to sleep for 3, 7, or 10 hours (IRR + S groups), or after they were kept awake with exposure to novel objects for 3, 7, or 10 hours (IRR + SD groups). After exploration only a few cells contain DSBs, and usually we counted only 1–2 foci in each nucleus. After irradiation instead, as expected, nearly all cells showed variable levels of DSBs, and thus we developed an automatic unbiased algorithm to quantify DSBs based on the overall mean γH2AX fluorescence as well as on the number of detectable discrete γH2AX foci (local maxima) within the cell nuclei (see Methods for details).

Within 1 hour post-irradiation the large majority of brain cells displaying DSBs were neurons (i.e. positive for the neuronal nuclear marker NeuN, Fig. 4b). The γH2AX fluorescence of putative non-neuronal cells (identified based on their shape and small size of their nuclei, <20 μm^2^) was manually annotated in a subset of IRR microscopic fields and accounted for only 3–5% of the overall γH2AX fluorescence, a very weak signal indistinguishable from the background (Suppl. Fig. 1). Relative to non-irradiated mice (C), IRR mice showed an increase in mean γH2AX fluorescence levels (~2.5 fold, p < 0.001) as well as in the number of local maxima (~6.5 fold, p < 0.001) (Fig. 4b–d). We also found that when compared to controls, male mice frequently displayed higher values of mean fluorescence (~3 fold, p < 0.001) and number of local maxima (~7 fold, p < 0.001) than age-paired females (fluorescence: ~2 fold, p < 0.001; maxima: ~6 fold, p < 0.001). This difference remained significant when post-irradiation values for males and females were directly compared to each other (fluorescence: p = 0.012; maxima: p = 0.027, Fig. 4c–e).

**Figure 5:**
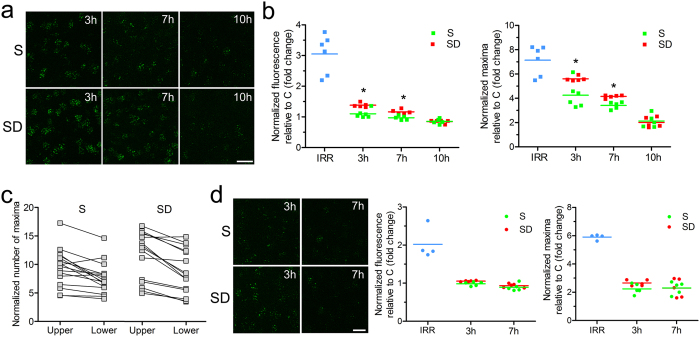
Differential effects of sleep on irradiation-induced DSBs in male and female mice. (**a**) Representative microscopic fields from the frontal cortex of post-irradiated sleeping (S) and sleep deprived (SD) male mice killed at different times (3 h, 7 h, 10 h) after light onset. Scale bar = 20 μm. (**b**) Left and right panels, quantification of γH2AX normalized fluorescence (middle) and maxima (right) in post-irradiated S and SD male mice sacrificed 3 h, 7 h, and 10 h after light onset. *p < 0.05 (**c**) quantification of γH2AX maxima from the upper and lower layers of the frontal cortex of S and SD male mice. (**d**) Left panel, representative microscopic fields from the frontal cortex of post-irradiated S and SD female mice killed 3 h and 7 h after light onset. Scale bar = 20 μm. Middle and right panels, quantification of γH2AX fluorescence (middle) and maxima (right) in post-irradiated S and SD female mice killed 3 h and 7 h after light onset.

To assess the effects of sleep on DNA repair we applied a two-ANOVA analysis using time and behavioral state as between factors. In male mice, we considered 3, 7, and 10 hours of sleep or exploratory wake as time points (Fig. 5a). Statistical analysis showed an effect of both time (mean fluorescence: F_2,25_ = 47.67, p < 0.0001; local maxima: F_2,25_ = 67.16, p < 0.0001) and behavioral state (mean fluorescence: F_1,25_ = 21.57, p < 0.0001; local maxima: F_1,25_ = 10.24, p = 0.0037), indicating a progressive decrease of DSBs levels with the passage of time, and a difference between sleep and wake, respectively. Since interaction was also significant (mean fluorescence: F_2,25_ = 6.67, p = 0.0048; local maxima: F_2,25_ = 4.34, p = 0.024), we performed post-hoc analysis and found that mice that could sleep post-irradiation showed significantly less DSBs than mice that were kept awake for 3 hours (mean fluorescence: t-test, p = 0.0023, % difference: ~25%; local maxima: t-test, p = 0.02, % difference: ~32%) or 7 hours (mean fluorescence: t-test, p = 0.0086, % difference: ~18%; local maxima: t-test, p = 0.0008, % difference: ~22%) (Fig. 5b). Note that although mice were kept awake using novel objects, the few DSBs that were presumably formed because of exploration cannot account for the difference between IRR + S and IRR + SD mice, because the number of exploration-related DSBs is more than three orders of magnitude smaller than the number of DSBs caused by irradiation. After 10 hours instead, DSBs levels were no longer different between mice that slept or stayed awake (mean fluorescence: t-test, p = 0.89, % difference: ~1%; local maxima: t-test, p = 0.71, % difference: ~6%; Fig. 5b), suggesting that early sleep has a more pronounced effect on DSBs repair than late sleep. Further analysis using the cortical layers as a within factor in a repeated measures three-way ANOVA confirmed the difference between sleep and wake (mean fluorescence: F_1,25_ = 20.68, p < 0.0001; local maxima: F_1,25_ = 11.48, p = 0.002), and revealed that superficial layers overall expressed higher levels of DSBs than deep layers (mean fluorescence: F_1,25_ = 5.075, p = 0.033; local maxima: F_1,25_ = 68.72, p < 0.0001, Fig. 5c). Since the analysis was performed on values normalized for cellularity (see Methods), we can exclude that these results simply reflect different cell density across layers.

In female mice, only the first 2 time points were considered, 3 and 7 hours. Statistical analysis showed an effect of time only for mean fluorescence values (F_1,16_ = 13.57, p = 0.002; maxima: F_2,25_ = 0.56, p = 0.47), while no effects of behavioral state were found (mean fluorescence: F_1,16_ = 2.17, p = 0.16; local maxima: F_1,16_ = 1.07, p = 0.32) (Fig. 5d). Breakdown analysis for different layers confirmed that as in males, superficial layers had more DSBs than deep layers (repeated measures three-way ANOVA: effect of layer: F_1,16_ = 6.83, p = 0.019 for fluorescence and F_1,16_ = 29.58, p < 0.0001 for maxima).

### Sleep is associated with increased expression of genes related to DSBs repair

**Figure 6:**
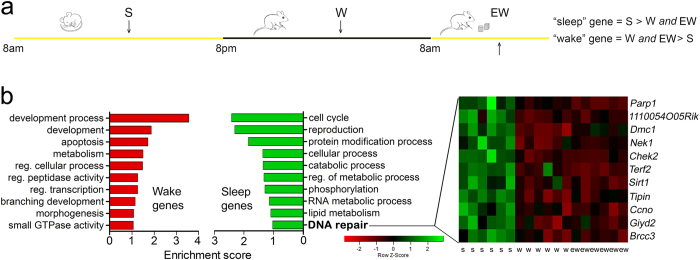
DNA repair genes upregulated in mouse forebrain after sleep. **(a)** Experimental groups used to identify sleep and wake genes. (**b**) Left panel, functional characterization of genes differentially expressed in wake (W + EW > S) and sleep (S > W + EW). A total of 392 unique genes for W + EW and 469 unique genes for S were identified and mapped for functional annotation analysis (DAVID default settings, except for the final group = 5 and multiple linkage threshold = 0.75). Top 10 functional annotation clusters in order of enrichment score are shown for W + EW and S. Right, heat diagram shows the probe set intensity of the DNA repair cluster for each individual S, W, and EW mouse.

Since the results so far showed that the decline in DSBs number was accelerated by sleep, we then asked whether genes involved in the DNA repair pathways are specifically upregulated during sleep relative to wake. To this end, we interrogated an unpublished gene dataset that we previously obtained in the context of a study to identify oligodendrocyte genes modulated by behavioral state (see Methods for details). Note that the method that we used, translating ribosome affinity purification (TRAP) technology combined with microarray analysis, involves cell-specific expression of an EGFP-L10a ribosomal transgene to tag polysomes and immunoaffinity purify mRNAs. TRAP therefore allows the isolation of mRNAs attached to ribosomes, on their way to become proteins. The unpublished dataset was obtained using the “unbound” fractions (containing neurons and glia) from the forebrain region of sleeping mice (S, 6–7 h of sleep during the light phase; n = 6), spontaneously awake mice (W, 6–7 h of spontaneous wake at night; n = 6), and mice kept awake during the day through exposure to novel objects (EW, 4 h of forced enriched wake during the light phase, n = 6). We identified 987 probe sets differentially expressed because of the sleep/wake cycle (2.2% of 45101 probe sets; false discovery rate = 1%), representing 861 unique genes, including 469 sleep genes and 392 wake genes. As before^31^, “sleep” genes were defined as those expressed at higher levels in the sleep group relative to both wake groups, whereas “wake” genes were those with higher expression in both spontaneous and forced enriched wake than in sleep. Thus sleep and wake genes as defined here reflect changes resulting from behavioral state, without confounding effects due to time of day or exposure to light. In addition, since by definition “wake” genes had higher levels of expression in both spontaneously awake mice and mice kept awake with novel objects, confounding effects such as stress were minimized. Sleep and wake genes were clustered to distinct categories using the gene annotation enrichment analysis provided by the Database for Annotation, Visualization and Integrated Discovery (DAVID v6.7). As expected, state-dependent genes belonged to different functional categories, including apoptosis for wake genes and lipid metabolism for sleep genes. Interestingly, among the sleep genes there was also an enriched cluster related to DNA repair that included *Brcc3*, *Tipin*, *Chek2*, *Nek1*, *Parp1*, and *Sirt1* as the most representative genes (Fig. 6). *Tipin* had previously been identified as “sleep” gene also in another study^32^. Since exploration of novel objects is associated with higher levels of DSBs than forced wake on a treadmill, we also performed a pair-wise comparison between W and EW. We found 2879 differentially expressed genes, including 1852 upregulated in EW and 1027 upregulated in W. A cluster of genes involved in DNA damage and repair was observed in EW (Enrichment Score: 1.37; Suppl. Table 1), but not in W. Of these genes, some were related to cellular stress and apoptosis (*Casp3, Hspa1b, Aen*) and others were associated with DNA damage and repair (*Trp53bp1, Fanci, Chek1*). *Trp53bp1* codes for the damage response factor 53BP1, which is involved in the regulation of the NHEJ/HR pathway choice^33^. *Fanci* is mainly implicated in the repair of DNA inter-strand cross-links, a type of DNA damage that forms a transient DSB and occurs during the S-phase^34^, while *Chk1* is involved in the cell cycle arrest in response to various types of genotoxic stresses^35^. Overall, these results suggest that wake with exploration, relative to spontaneous wake, may lead to the specific activation of cellular pathways involved in DNA damage and repair. It is worth noting, however, that the differential gene expression between W and EW may also depend on differences in circadian time and/or light exposure, as EW and W were collected at different times of day.

## Discussion

In this study we built on previous results showing that exploratory activity causes an increase in DSBs in mice cortical neurons^20^. In both flies and mice, we found that the occurrence of DSBs occurs when wake is associated with conditions that favor learning and synaptic plasticity, such as exploration and exposure to an enriched environment, while more automatic behaviors – mice moving on a treadmill or flies housed in isolation inside small glass tubes - results in little, if any, formation of DSBs. These DSBs are rare in mouse frontal cortex, occurring only in a minority of pyramidal neurons, and mainly in deep layers. They likely represent signs of wake-dependent transcriptional activation, as suggested by recent studies showing that DSBs are transiently produced during the transcription of stimulus-induced genes, including plasticity-related immediate early genes *Fos, Jun, Npas4* and *Egr1/Zif268/NGFI-A*^21^,^22^. These DSBS occur only in the promoter region or inside the transcribed unit of the gene, likely to relieve topological constrains that limit transcriptional initiation^21^ and elongation^22^. Of note, one of the mechanisms that underlies the widespread changes in cortical gene expression between sleep and wake is the activity of the noradrenergic system of the locus coeruleus (LC), whose neurons project diffusely over the entire brain and are active during wake, especially in response to salient events, but not during sleep^36^. During wake, rats subjected to LC lesions to deplete the brain of norepinephrine show a marked decrease in the expression of plasticity-related genes, including those coding for Fos, Arc, BDNF, and NGFI-A, as well as a blunted homeostatic response to sleep deprivation, suggesting a reduced need for sleep^37^,^38^,^39^. By contrast, mice subjected to adrenalectomy, which abolishes the increase in corticosterone levels often seen during acute sleep deprivation, still show strong induction of plasticity-related genes during wake, and their sleep homeostatic response is maintained^32^. Thus, DSBs linked to exploration and learning may represent one of the “costs” of wake-related plasticity, facilitating gene transcription in the short term^31^,^40^,^41^, but potentially increasing the long-term risk of mutations.

DSBs due to exploration are assumed to be steadily repaired through an error-free, tyrosyl DNA phosphodiesterase 2 (TDP2)-dependent mechanism of Non-Homologous End Joining (NHEJ)^21^,^42^, and possibly through ataxia telangiectasia mutated protein (ATM)-dependent NHEJ, a potentially more mutagenic pathway that requires the pre-processing of DNA ends by nucleases^43^. The phosphorylation of histone protein H2A, the marker that we used to detect DSBs, occurs within minutes from the application of DNA-damaging agents including glutamate receptor agonists, and may require >24 hours to return to baseline levels^29^. Thus, the repair starts already in wake, and the DSBs seen in awake animals may represent those foci formed in the very few hours before the brains were collected. In mice however, the number of DSBs remained the same after 6 and 12 hours of wake, either because the DSBs formed during exploration could not be repaired until sleep occurred, or because in the last 6 hours of wake DSBs were formed at a much lower rate that could match the repair rate. This second possibility seems more plausible and in line with the results of the irradiation experiment, where we found that both sleeping and sleep deprived animals were able to repair irradiation-induced DSBs, but both sleeping male mice and female flies approached baseline levels of DSBs more rapidly than sleep deprived animals. Of note, irradiation-induced DSBs are not generated through a topoisomerase-dependent mechanism, are not confined within specific gene boundaries, and their repair relies more on ATM-dependent NHEJ than their transcription-associated counterpart^44^, as they require more extensive DNA-ends pre-processing^45^. Thus, sleep may boost DNA repair through different pathways, and may do so for several reasons. Since neurons are more hyperpolarized during sleep and tend to fire less, sleep may generally provide a time when more energy can be devoted to housekeeping functions, including DNA repair, akin to how sleep seems to boost protein synthesis without being absolutely required for it^4^,^46^. In the case of exploration, another general advantage provided by sleep may be the fact that the very cause of DSBs, the induction of plasticity-related genes, is low in sleep relative to wake. There may also exist specific mechanisms through which sleep promotes the repair of DSBs, as suggested by our array data, since we found that in the mouse forebrain several genes involved in DNA repair are expressed at higher levels in sleep than in wake. This cluster included *Brcc3*, *Tipin*, *Chek2*, *Nek1*, *Parp1*, and *Sirt1* as the most representative genes. Notably, all of these genes are involved in the repair of DSBs and are important for post-transcriptional enhancement of the NHEJ pathway (*Brcc3*, *Nek1*, *Sirt1*)^47^,^48^,^49^,^50^,^51^, for the recognition of DSBs (*Parp1*)^52^, and for the recruitment of proteins that are involved in the early stages of DSB repair (*Chek2*, *Brcc3*, *Parp1*, *Tipin*)^53^,^54^.

In mice, the post-irradiation beneficial effect of sleep was confined to males, since in females 3 and 7 hours after the insult DSBs declined to the same extent in sleeping and sleep deprived individuals. However, females were in general considerably faster in repairing DNA damage than males, and this may have prevented us from finding any sleep-dependent effect, at least at the time points that we studied. For instance, 3 hours after irradiation the number of foci was <3 in both sleeping and sleep deprived females, ~4 in sleeping males, and >5 in sleep deprived males (Fig. 5). Amyloid-β, a hallmark of Alzheimer disease that was shown to increase the level of exploration-related DSBs^20^, exerts stronger toxic effects on mitochondria of young male mice compared to young females^55^. Moreover, estrogens protect mitochondria from oxidative stress^56^, although the latter usually leads to DNA single strand breaks, not DSBs. Perhaps more crucially, estrogen-receptor-alpha stimulates the transcription of DNA-PK, a key enzyme of the NHEJ pathway, and increases DSBs repair speed^57^,^58^.

We found that exploration and gamma irradiation affected different cortical layers. Following exploration, deep cortical layers were more prone to develop DSBs than superficial layers. One reason may be the higher firing rate, and thus the higher cellular activity, of layer V neurons as compared to neurons in superficial layers^59^. Indeed, levels of DSBs increase in the relevant areas after sensory or optogenetic stimulation^20^, suggesting that the induction of DSBs is at least partially linked to an increase in cellular activity. By contrast, after gamma irradiation we found more DSBs in superficial than in deep layers, perhaps because the irradiation dose declines significantly as it penetrates into the brain tissue.

A limitation of this study is that we relied on one single marker, the phosphorylation of a variant form of histone H2A, to detect DSBs. This is a sensitive and well-established method, considered as the gold standard in DSB quantification^29^, but it provides an indirect measure of DSBs and can lead to false positives^60^. However, the detection of phosphorylated H2A foci yields results consistent with those obtained with techniques that more directly assess DSBs, such as the comet assay^20^,^29^,^61^,^62^, and in fact, it is sometimes more sensitive than these methods^29^,^62^. Nonetheless, future studies should use additional markers of DSBs, as well as markers for other types of DNA damage.

Deficiencies in the cellular response to DNA damage lead to neurodegeneration and cancer^63^, and epidemiological studies have linked sleep disordered breathing to cancer, although the association remains tentative and the role of sleep loss per se, as compared to hypoxia, is unclear^64^. Similarly, a link between shift work and cancer has been found, but the contribution of circadian disruption as compared to sleep disruption is unknown^65^. A recent study found increased oxidative DNA damage in liver, lung and small intestine after 10 days of sleep loss but neuronal effects were not studied^66^. Our results using very short periods of sleep loss suggest a nonessential but facilitatory role of sleep in the repair of DSBs in the brain, supporting the view that sleep disruption per se may be detrimental for DNA repair in many tissues.

## Materials and Methods

### Mice experiments

#### Animals

C57BL/6J mice of 12 weeks of age of either sex were used for this study. All animal procedures and experimental protocols followed the National Institutes of Health Guide for the Care and Use of Laboratory Animals and were approved by the licensing committee. All animal facilities were reviewed and approved by the institutional animal care and use committee (IACUC) of the University of Wisconsin-Madison, and were inspected and accredited by association for assessment and accreditation of laboratory animal care (AAALAC).

#### Video monitoring of sleep and wake

Three days before the experiment, mice were housed individually in transparent Plexiglas cages in dedicated boxes with a 12 h light − 12 h dark cycle (lights on at 8 am) at 23 °C with access to food and water ad libitum, and constantly monitored with infrared cameras (OptiView Technologies). Sleep and wake were estimated based on motor activity and quantified using custom-made video-based motion detection algorithms, as previously described^67^. When compared to electroencephalographic recordings, this method consistently estimates total sleep time with ≥90% accuracy^68^.

#### Sleep and exploration experiments

For this part of the study five experimental groups were used: 1) sleep mice (S, n = 6) were allowed to sleep for 6 h; 2) Exploration mice (E, n = 8) were engaged in active exploration using novel objects for 6 h; 3) Exploration + Sleep mice (E + S, n = 6) actively explored novel objects for 6 h, followed by 6 h of recovery sleep; 4) Exploration + Treadmill mice (E + T, n = 6), actively explored novel objects for 6 h, followed by 6 h of forced walking with little or no exploration on a slowly rotating treadmill; 5) Treadmill mice (T, n = 6), 6 h of forced walking with little or no exploration on a slowly rotating treadmill. All the experiments started at light onset (8 am).

#### Irradiation experiments

Three days before the experiment mice were housed in individual cages and monitored as described before. The day of the experiment mice were transferred to the irradiation facility at ~7 am, about one hour before light onset. During the transfer, cages were placed in a dark box. They were irradiated on a rotating platform in a Shepherd Mark I irradiator with a cesium source with a non-lethal dose of 1000 rad (irradiation time ~5 min), and transferred back to their own cages by 8 am (light on). Mice were then either left undisturbed (S) for 3 h (n = 11), 7 h (n = 10), or 10 h (n = 5), or kept awake (SD) with novel objects for 3 h (n = 10), 7 h (n = 10), or 10 h (n = 5). Overall, during the 10 h following irradiation, mice showed a motion activity pattern comparable to that seen in the same animals the day before, suggesting that sleep was not acutely affected by irradiation (sleep before irradiation: 79.6 ± 2.3%; after irradiation: 74.5 ± 16.5%; p = 0.56). However, during the first hour post-irradiation mice were awake longer than during baseline, likely because “excited” by the transfer from the irradiation facility back into their cages. Thus, the 3 h S group was asleep mainly during the last 2 of the 3 hours. Two other groups of mice were also used: “control” mice (C, n = 8) were left undisturbed in their cages and killed towards the end of the light phase (~6 pm), and post-irradiation mice (IRR, n = 10), whose brains were collected ~1 h after irradiation.

#### Immunohistochemistry

Mice were perfused with 4% paraformaldehyde; brains were post fixed in the same fixative for 24 h and cut using a vibratome (50 μm sections). Three coronal sections of frontal cortex (AP: 0.1–0.2 mm from Bregma) for each mouse were rinsed with NGS 10% + 0.03% Triton for 1 h, then incubated overnight at 4 °C with a primary antibody against the phosphorylated histone protein H2A variant X at serine 139 (γH2AX, 1:1000, Millipore), a well-established marker of DSBs^29^. After washes in PBS, sections were rinsed with a secondary antibody (Alexa fluor 488-conjugated goat anti-rabbit IgG, Molecular Probes), counter-stained with propidium iodide (PI) to identify cell nuclei, and examined with a confocal microscope (Prairie Technologies).

#### Image acquisition and analysis

For each section of frontal cortex, z-stacks (z-step: 0.75 μm, 36 images per stack) of 15–20 fields of layers II-III and V-VI were randomly imaged using a UPlan FL N x40 objective (numerical aperture 1.3, pixel size: 581 nm). For sections of groups S, E, E+S, E+T, T cells were visually scored by an operator blind to the experimental condition, and cells containing at least one γH2AX-positive focus in the nucleus were scored as positive. In the irradiation experiments nearly all cells showed variable levels of DSBs, making manual counting of positive cells impracticable. Thus, γH2AX fluorescence was quantified using a custom made algorithm in FIJI. Briefly, stacks of both channels were collapsed on two separate Z-projections, using the points of maximum intensity. The PI-stained Z-projections were then filtered with the Gaussian Blur method (radius = 3) and binarized with the Auto-Threshold function (method = default). These processed images constituted the nuclear mask and their average intensity (i.e. the percentage of the area of the image that was positive after applying the threshold) was computed as a measure of cellularity. Using Image Calculator (function AND), an intersection between the nuclear masks and the Z-projections of the images with γH2AX fluorescence was then created. Next, the average intensity of the γH2AX fluorescence was computed, as well as the number of local maxima (function: find maxima, noise tolerance: 50) roughly corresponding to the γH2AX foci. For the statistical analysis, both mean fluorescence and maxima values were normalized to the averaged intensity of nuclei (cellularity).

#### Array analysis

We used the array data available at NCBI GEO database (GSE48369) to perform gene expression analysis of forebrain samples collected from sleeping (6–7 h of sleep during the light phase), awake (6–7 h of spontaneous wake at night), and forced enriched wake (4 h of sleep deprivation through exposure to novel objects during the light phase) mice. For detailed methods see ref. 67. Briefly, samples (six for each behavioral state) were collected using the genetically targeted translating ribosome affinity purification (TRAP) methodology from bacterial artificial chromosome (BAC) transgenic mice expressing EGFP tagged ribosomal protein L10a in oligodendrocytes. For each animal, one forebrain sample was immediately processed, and immunoprecipitated to isolate oligodendrocytes. The precipitated portion formed the bound sample (IP) enriched in oligodendrocytes and the remaining part formed the unbound sample (UB) enriched in all the remaining cell types (neurons and other glia cells). Then, both IP and UB samples were processed, RNA was extracted, and run on Affymetrix GeneChip Mouse Genome 430 2.0 arrays. In the present study, we used array data obtained from the UB samples, which had been processed but never analyzed for comparative analysis before. Data were normalized within each behavioral state group using Robust Multiarray Average. To identify transcripts that were differentially expressed across behavioral states, comparisons were carried out using the Welch’s t test with Benjamini and Hochberg FDR multiple-test correction. All probe sets with fold change >30% and p < 0.01 were selected for cluster analysis.

#### Statistical analysis

Parametric statistic was used as the distribution of data passed the normality test (Kolmogorov-Smirnov test). Alpha was set to 0.05.

### Drosophila experiments

#### Fly Husbandry

Male w[1118]; P{w[+mC] =UAS-GFP.nls}8 flies were crossed to w[1118]; nSyb-Gal4 females to generate progeny expressing green fluorescent protein (GFP) that accumulates in the nucleus. Female progeny were harvested within 12 h after eclosure and kept in Drosophila activity monitor (DAM) glass tubes unless otherwise stated. Parents and progeny were cultured with standard cornmeal molasses and maintained and tested at 20 °C temperature, 68% humidity, on a 12 h:12 h light:dark cycle.

#### Sleep/wake recordings and sleep deprivation

At the beginning of the experiment, single flies were placed in the DAM glass tubes with enough food for 1 week of recording. Monitors were housed inside environmental chambers where temperature and humidity were kept constant (20 °C, 68%). Data analysis was performed using custom-designed software developed in our laboratory. Sleep and wake were determined for consecutive 1-min epochs according to standard published criteria^15^,^25^. Wake was defined as any period of at least 1 min characterized by activity (≥1 count/min), and sleep was defined as any period of uninterrupted behavioral immobility (0 counts/min) lasting >5 min, which is associated with increased arousal threshold. Sleep deprivation through mechanical stimulation was performed as before^25^. In the experiment using enriched wake, flies were housed inside a tube large enough (70 cm long, 10 cm in diameter) to allow flight and social interactions, as confirmed with video recordings^27^. Note that when multiple flies are housed in these large tubes, monitoring individual fly activity is not possible, but previous experiments^27^ as well as direct observation and video recordings confirmed that female flies remain predominately awake during the light period.

#### Irradiation experiments

The day of the experiment groups of female flies (~20) were placed in a standard plastic vial with cornmeal molasses food extending 1 cm from the base and transferred to the irradiation facility in the afternoon. During the transfer, flies were placed in a dark box. They were irradiated with non-lethal doses of 2500 rad in a Shepherd Mark I irradiator with a cesium source (~7 min of irradiation time), and transferred back to their DAM tubes or harvested to test for DSB frequency (IRR group). One group of flies was then allowed to sleep until the next morning (IRR + S, n = 11), while another group was sleep deprived through mechanical stimulation starting ~ one hour before lights off, and for the entire night (IRR + SD, n = 6). A non-irradiated control group (C, n = 10) was also harvested in the afternoon at the same time as the IRR group, after having been awake most of the light period as the IRR flies.

#### Immunohistochemistry

Flies were immediately drowned in cold PBST. Brains were detached from the cuticle and left attached to the body. Paraformaldehyde (Electron Microscopes) was added to 4% concentration and fixation allowed to occur over 20 min with gentle shaking. Samples were then rinsed with PBST, incubated overnight at 4 °C with normal goat serum, and then reacted with a primary mouse antibody against gamma-H2AV (UNC93-5.2.1, 1:10 supernatant, DSHB;) and a rabbit antibody against GFP (A21311, 1:1000, Invitrogen). Secondary staining occurred with goat anti-mouse antibody labeled with 568 (A11004, Invitrogen), and goat anti-rabbit antibody labeled with 488 alexa fluor. Incubation time for all antibodies was 2 days at 4 °C in PBST. In preparation for imaging, individual brains were placed in 8-well seal spacer (S24737, Invitrogen) containing hard mount Vectashield. Both posterior and anterior sections of each brain were, sandwiched between coverslips, were imaged with a confocal microscope (Prairie Technologies).

#### Image processing

Using a custom built graphical user interface designed in MATLAB, regions of interest (ROI) were manually selected. Although GFP staining was enriched in the nuclear region of neurons, as expected, it was also evident in the cytoplasm, making it very difficult to identify the nuclei based on this marker alone. Fortunately, however, the antibody against phosphorylated H2AV consistently produced a low intensity background signal in the nuclear regions. Thus, we identified as nuclei only those regions in which both the GFP and the H2AV background staining exceeded a user-selected threshold, and the signals produced by both markers were combined to generate a 3-dimensional mask specific to nuclear regions. Further processing in Matlab filled in holes and counted the number of nuclei within each region. Sites of intense H2AV staining at double strand breaks (H2AV foci) were identified by dividing H2AV fluorescence by the consistent background staining produced by H2AV within the nuclear region. Background fluorescence was determined by eroding intense foci less than 6 um wide and then dilating the remaining background staining pattern. Mean number of nuclei counted across the brain was 2786 ± 183 nuclei/animal. The number of cells did not significantly differ between groups in any of the experiments (Kruskal-Wallis test, p > 0.05). The use of the ratio - H2AV to background H2AV - allowed us to reduce possible technical sources of variability across individual flies, including differences during the staining of the samples or during image acquisition. Foci were counted as H2AV positive when their intensity was 30 times greater than background and their volume was >0.2 um^3^. This method consistently found more foci in irradiated compared to untreated animals.

#### Statistical analysis

Comparisons with 3 or more groups were first tested with the Kruskal-Wallis test to determine whether any one group was significantly different (p < 0.05). Post-hoc comparison between samples was made with the Mann-Whitney U-test (p < 0.05).

## Additional Information

**How to cite this article**: Bellesi, M. *et al*. Contribution of sleep to the repair of neuronal DNA double-strand breaks: evidence from flies and mice. *Sci. Rep*. **6**, 36804; doi: 10.1038/srep36804 (2016).

**Publisher’s note:** Springer Nature remains neutral with regard to jurisdictional claims in published maps and institutional affiliations.

## Supplementary Material

Supplementary Information
