# Supplementary material for: Contribution of sleep to the repair of neuronal DNA double-strand breaks: evidence from flies and mice
Source: Sci Rep. 2016 Nov 10;6:36804. doi: 10.1038/srep36804 (PMC5103291; doi:10.1038/srep36804)
Supplement: Supplementary Information [file srep36804-s1.pdf]

Contribution of sleep to the repair of neuronal DNA double-strand breaks: evidence from flies and mice

Michele Bellesi[1,2#], Daniel Bushey[1#], Mattia Chini[1], Giulio Tononi[1] and Chiara Cirelli[1*]

[1] Department of Psychiatry, University of Wisconsin-Madison, 6001 Research Park Blvd, Madison, WI 53719 USA
[2] Department of Experimental and Clinical Medicine, Section of Neuroscience and Cell Biology, Università Politecnica delle Marche, Ancona, Italy

#, co-first authors

* Correspondence:
Chiara Cirelli, MD PhD
Department of Psychiatry, University of Wisconsin - Madison
6001 Research Park Blvd
53719 Madison, Wisconsin, USA
ccirelli@wisc.edu

Supplementary Figure 1 | Effects of gamma-irradiation on DSBs in non-neuronal cells. a, Representative example of a cortical field from an IRR male mouse. Manually segmented non-neuronal cells are depicted in orange (left panel). PI staining (blue) and γH2AX fluorescence (green) are represented in the middle and right panel, respectively. Arrow indicates a non-neuronal cell that shows weak γH2AX fluorescence (green) in the right panel. Scale bar: 5um. **b**, Averaged ratio (in %) between the γH2AX fluorescence of manually segmented non-neuronal cells and the whole (non-neuronal+neuronal) γH2AX fluorescence in IRR male mice (n=6).

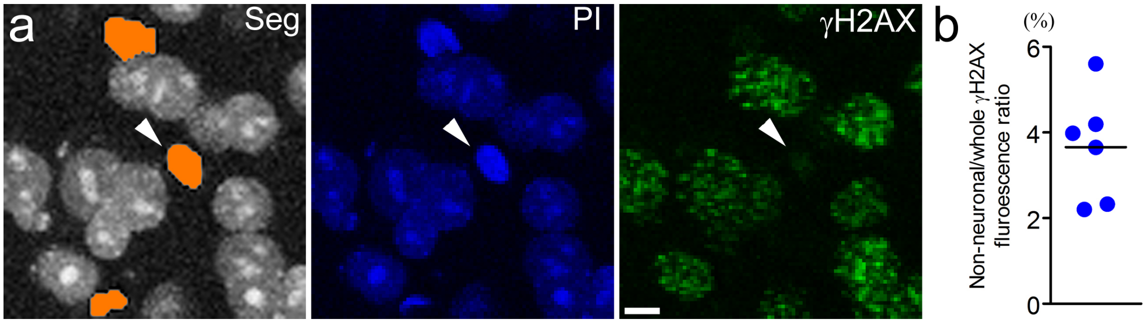

Supplementary Table 1 | Top functional annotation clusters with enrichment score > 1 for W and EW groups.

Up-regulated in W

Cluster	Enrichment Score
Development	3
Cell differentation	2.3
Reproduction	2
Nervous system development	1.9
Regulation of transcription	1.9
Morphogenesis	1.6
Transcription	1.6
Cell development	1.5
Regulation of cell development	1.5
Chromosome organization	1.4
Cell development	1.4
Immune cell activation	1.2
Immune system activation	1.6
Phosphorylation	1.2
Regulation of development	1
MAPKKK cascade	1
Regulation of kinase cascade	1
Methylation	1

Up-regulated in EW

Cluster	Enrichment Score
Development	5.1
Regulation of cellular process	3.8
Regulation of biological process	3.7
Tissue development	3.3
Metabolism	3.2
Metabolic process	3
Apoptosis	2.4
Reproduction	2.3
Catabolic process	2.2
Cell development	1.9
Tissue development	1.8
Regulation of immune system	1.7
Immune system activation	1.6
Regulation of immune system	1.6
Regulation of phosphorylation	1.6
Synaptic transmission	1.5
Reproductive process	1.5
Cell cycle	1.42
Kinase activity	1.4
**Response to stress/DNA damage**	**1.4**
Axonogenesis	1.4
Morphogenesis	1.2
Cell migration	1.2
Development	1.2
Cell motion	1.1
Cell activation	1.1
Cell differentation	1.1
Cell morphogenesis	1
